# Efecto de la hipomineralización incisivo molar en la calidad de vida relacionada con la salud bucal de niños y adolescentes: una revisión sistemática

**DOI:** 10.21142/2523-2754-1004-2022-130

**Published:** 2023-12-26

**Authors:** Camilo Romo Pérez, Luis Lobo Cortés, María José Morales Rojas, Alma Luz San Martín López, Karem Guadalupe Ramírez Vera

**Affiliations:** 1 Unidad de Investigación en Medicina Estomatológica Preventiva y Social (UIMEPS), Universidad del Magdalena. Santa Marta, Colombia. camiloromoap@unimagdalena.edu.co, luisloboac@unimagdalena.edu.co Universidad del Magdalena Unidad de Investigación en Medicina Estomatológica Preventiva y Social (UIMEPS) Universidad del Magdalena Santa Marta Colombia camiloromoap@unimagdalena.edu.co luisloboac@unimagdalena.edu.co; 2 Programa de Odontología, Universidad del Magdalena. Santa Marta, Colombia. mariamoralesjr@unimagdalena.edu.co Universidad del Magdalena Programa de Odontología Universidad del Magdalena Santa Marta Colombia mariamoralesjr@unimagdalena.edu.co; 3 Facultad de Odontología, Universidad Veracruzana, Poza Rica, México. alsanmartin@uv.mx, kramirez@uv.mx Universidad Veracruzana Facultad de Odontología Universidad Veracruzana Poza Rica Mexico alsanmartin@uv.mx kramirez@uv.mx

**Keywords:** hipomineralización incisivo molar, calidad de vida, salud infantil, evaluación del resultado de la atención al paciente, molar incisor hypomineralization, quality of life, child health, patient outcome assessment

## Abstract

**Introducción:**

La hipersensibilidad, alteración del aspecto dental y fracturas son problemas comunes en la hipomineralización incisivo molar (HIM) que generan problemas funcionales y socioemocionales.

**Objetivo:**

evaluar el efecto de la HIM en la calidad de vida relacionada con la salud bucal de niños y adolescentes.

**Materiales y métodos:**

Se realizó una búsqueda bibliográfica sistemática en bases de datos electrónicas (PubMed, Epistemonikos, Dentistry & Oral Sciences Source y Biblioteca Virtual en Salud). Se identificaron estudios observacionales en inglés o español realizados entre 2016-2022 que evaluaron la calidad de vida de niños y adolescentes con hipomineralización incisivo molar. La mayoría de los estudios fueron de buena calidad metodológica.

**Resultados:**

De 96 estudios identificados, trece fueron incluidos en la síntesis. El criterio diagnóstico para hipomineralización incisivo molar más frecuente fue European Academy of Paediatric Dentistry index y nueve estudios informaron sobre la gravedad de la enfermedad. La escala más usada para medir la calidad de vida fue Child Perception Questionnaire (CPQ). Según la percepción de los niños, las dimensiones más afectadas fueron “Síntomas orales” y “Bienestar emocional”, mientras que, según los padres, fueron “Síntomas orales” y “Limitación funcional”. Las niñas con hipomineralización incisivo molar tuvieron peor calidad de vida relacionada con la salud bucal.

**Conclusiones:**

El efecto negativo de la hipomineralización incisivo molar en la calidad de vida relacionada con la salud bucal infantil parece variar entre la percepción de los padres y de los niños.

## Abstract (en)

**Introduction:**

Hypersensitivity, altered dental appearance and fractures are common problems in molar incisor hypomineralization that generate functional and socio-emotional problems.

**Objective:**

to evaluate the effect of MIH on oral health-related quality of life in children and adolescents.

**Materials and methods:**

A systematic bibliographic search was carried out in electronic databases (Pubmed, Epistemonikos, Dentistry & Oral Sciences Source and Virtual Health Library). Observational studies in English or Spanish conducted between 2016-2022 that evaluated the quality of life of children and adolescents with molar incisor hypomineralization were identified. Most studies were of good methodological quality.

**Results:**

Of 96 identified studies, thirteen were included in the synthesis. The most frequent diagnostic criterion for hypomineralization of molar incisors was the index of the European Academy of Pediatric Dentistry and nine studies reported the severity of the disease. The most widely used scale to measure quality of life was the Child Perception Questionnaire (CPQ). According to the children's perception, the most affected dimensions were "Oral Symptoms" and "Emotional Well-Being", according to the parents they were "Oral Symptoms" and "Functional Limitations". Girls with molar incisor hypomineralization had worse oral health-related quality of life.

**Conclusions:**

The negative effect of molar incisor hypomineralization on children’s oral health-related quality of life seems to vary between the perception of parents and children.

## INTRODUCCIÓN

La hipomineralización incisivo molar (HIM) es una patología perteneciente al grupo de los llamados defectos del desarrollo del esmalte (DDE), considerada un defecto cualitativo del esmalte principalmente en primeros molares permanentes, aunque, también se pueden ver frecuentemente lesiones en incisivos permanentes superiores o inferiores. Asimismo, algunos estudios reportan algunos casos de HIM en caninos permanentes y en segundos molares temporales. Clínicamente, estas lesiones pueden presentarse como opacidades delimitadas de color blanco, cremoso, amarillo o marrón en los casos leves, y con cambios a nivel estructural del esmalte que lo hacen frágil, poroso, susceptible de fracturas y, posiblemente, con mayor riesgo de destrucción coronal y pérdida dental prematura ^1^^-^^4^.

La prevalencia reportada en estudios anteriores de esta patología en niños entre 6 y 12 años oscila entre el 7% y el 35,4%, según el país ^5^^-^^7^. Por otro lado, una revisión de la literatura indicó una prevalencia combinada de HIM del 14,2% a nivel mundial ^8^ lo que la convierte en un problema relevante de salud pública no solo por las cifras de la dinámica del fenómeno, que se cree que puede haber un gran subregistro de casos, sino también por sus efectos sobre el bienestar general de los menores. 

Desde hace más de dos décadas se habla de HIM, el interés de los investigadores en esta condición, sus factores asociados y consecuencias relacionadas como el impacto sobre la Calidad de Vida Relacionada con la Salud Bucal (CVRSB) ha aumentado. Los DDE, dependiendo de su severidad, alteran la estética de las personas, en esa perspectiva, los niños en etapa escolar comienzan a darle importancia a su apariencia, por lo que la insatisfacción personal, evitar sonreír, verse o sentirse diferentes, así como ser objeto de juicios o burlas por parte de otros niños por causa de las condiciones dentales, les puede generar impactos negativos en su bienestar emocional y social ^9^^,^^10^.

El componente físico y funcional de los menores con HIM se ve afectado principalmente por la hipersensibilidad que genera el esmalte poroso; de la misma manera, la pérdida dental temprana por condiciones graves de HIM afecta la oclusión dental y hace que el paciente requiera tratamiento ortodóntico ^11^^-^^13^. 

Abordar el tratamiento de la HIM es un reto clínico, puesto que es difícil lidiar con la sensibilidad dental; asimismo, es frecuente la desadaptación marginal de las restauraciones producto de la inadecuada adhesión entre el sustrato dental y el material restaurativo debido a la hipomineralización del sustrato dental ^14^.

Otros factores, como la ansiedad y el miedo por la consulta dental, aumentan en estos pacientes, posiblemente porque los niños con HIM reciben aproximadamente diez veces más intervenciones dentales que los niños sin la patología, lo que incrementa también los costos de la atención de la HIM ^11^^,^^16^. En antecedentes de estudios observacionales, se evidencia que los impactos sobre las dimensiones de la CVRSB parecen variar de acuerdo con la severidad de la HIM, de quien responde la escala y del instrumento utilizado ^16^^,^^17^. Sin embargo, es claro que la HIM afecta negativamente la CVRSB de niños y adolescentes. 

A pesar de que existen varios estudios primarios de CVRSB en pacientes con HIM, se evidencia una falta de intentos sistemáticos por resumir los hallazgos de este tipo de estudios, ya que, al momento de plantear este estudio, solo una revisión sistemática ha resumido la evidencia de un posible impacto negativo en la CVRSB de niños y adolescentes afectados con HIM, señalando un mayor deterioro de la CVRSB en estos pacientes ^18^. No obstante, esta revisión solo pudo incluir ocho artículos disponibles debido al periodo de realización del estudio, que fue hasta el 2020. Por otra parte, esta revisión abordó superficialmente la evaluación de la CVRSB según la severidad de HIM y su impacto individual sobre las dimensiones. Asimismo, no se informó de aspectos importantes del diagnóstico de HIM, como la calibración de los examinadores, ni del ajuste de los resultados por factores de confusión en el análisis estadístico. En consecuencia, es razonable pensar que los estudios nuevos pueden complementar una base de evidencia que respalde el uso de las diferentes escalas de medición de CVRSB en niños con HIM, contemplando no solo la presencia de la patología, sino también su severidad, la edad de la población afectada y la estructura de la escala del constructo que se pretende evaluar. Por lo tanto, el objetivo de esta revisión sistemática fue resumir la evidencia más reciente de la evaluación del efecto de la HIM y su severidad en las dimensiones de la CVRSB de niños y adolescentes.

## MATERIALES Y MÉTODOS

La presente revisión sistemática se realizó siguiendo las pautas Preferred Reporting Items for Systematic Reviews and Meta-Analyses (PRISMA) ^19^. El protocolo de revisión fue aprobado *a priori* por todos los autores.

### Definición de la pregunta de investigación

¿Cuál es el efecto de la hipomineralización incisivo molar sobre la calidad de vida relacionada con la salud bucal y sus dimensiones en niños y adolescentes, y si existe variación del efecto sobre las dimensiones de la Calidad de Vida Relacionada con la Salud Bucal de acuerdo con quien responda el instrumento?

### Estrategia PECOS

Población (P): Niños y adolescentes entre 6-17 años

Exposición (E): Diagnóstico de Hipomineralización Incisivo Molar (HIM)

Comparador (C): Sin diagnóstico de hipomineralización incisivo molar (HIM) o con otra condición bucal

Resultado (*Outcome* O): Calidad de Vida Relacionada con la Salud Bucal (CVRSB).

Diseño de estudio (*Study design* S): Estudios de cohorte, casos y controles, estudios antes-después (pre-post) con y sin grupo de control, y estudios transversales

### Estrategias de búsqueda bibliográfica

Se realizó una búsqueda de la literatura en cuatro bases de datos de ciencias de la salud: PubMed, Epistemonikos, Dentistry & Oral Sciences Source y Biblioteca Virtual en Salud (BVS) usando combinaciones de los siguientes términos de búsqueda en inglés: “*molar incisor hypomineralisation*” OR “*molar incisor hypomineralization*” OR “*molar-incisor hypomineralisation*” OR “*molar-incisor hypomineralization*” OR “MIH”, “*oral health-related quality of life*” OR “oral health related quality of life” OR “OHRQOL” OR “OHRQoL” y en español: “hipomineralización incisivo molar” OR “HIM” AND “calidad de vida relacionada con la salud oral” OR “calidad de vida relacionada con la salud bucal” OR “CVRSB” OR “CVRSO”. Se utilizó un filtro de fecha de publicación restringido desde 2016 hasta 2022 y por tipo de publicación para que únicamente se identificaran estudios primarios (Tabla 1). 

**Tabla 1:** Estrategias de búsqueda empleadas en la identificación de los estudios

Base de datos	Estrategia de búsqueda	N.^o^ Resultados
PubMed	((((((("Oral Health-Related Quality of Life"[Title/Abstract])) OR ("oral health-related quality of life"[Title/Abstract])) OR ("oral health related quality of life"[Title/Abstract])) OR ("OHRQoL"[Title/Abstract])) OR (“ohrqol”[Title/Abstract])) AND ("Molar-Incisor Hypomineralization"[Title/Abstract])) AND ("MIH"[Title/Abstract])	16
Epistemonikos	(title:((title:((title:(molar incisor hypomineralization) OR abstract:(molar incisor hypomineralization)) OR (title:(molar-incisor hypomineralization) OR abstract:(molar-incisor hypomineralization)) OR (title:(MIH) OR abstract:(MIH)) AND (title:(oral health-related quality of life) OR abstract:(oral health-related quality of life)) OR (title:(oral health related quality of life) OR abstract:(oral health related quality of life)) OR (title:(OHRQoL) OR abstract:(OHRQoL))) OR abstract:((title:(molar incisor hypomineralization) OR abstract:(molar incisor hypomineralization)) OR (title:(molar-incisor hypomineralization) OR abstract:(molar-incisor hypomineralization)) OR (title:(MIH) OR abstract:(MIH)) AND (title:(oral health-related quality of life) OR abstract:(oral health-related quality of life)) OR (title:(oral health related quality of life) OR abstract:(oral health related quality of life)) OR (title:(OHRQoL) OR abstract:(OHRQoL))))) OR abstract:((title:((title:(molar incisor hypomineralization) OR abstract:(molar incisor hypomineralization)) OR (title:(molar-incisor hypomineralization) OR abstract:(molar-incisor hypomineralization)) OR (title:(MIH) OR abstract:(MIH)) AND (title:(oral health-related quality of life) OR abstract:(oral health-related quality of life)) OR (title:(oral health related quality of life) OR abstract:(oral health related quality of life)) OR (title:(OHRQoL) OR abstract:(OHRQoL))) OR abstract:((title:(molar incisor hypomineralization) OR abstract:(molar incisor hypomineralization)) OR (title:(molar-incisor hypomineralization) OR abstract:(molar-incisor hypomineralization)) OR (title:(MIH) OR abstract:(MIH)) AND (title:(oral health-related quality of life) OR abstract:(oral health-related quality of life)) OR (title:(oral health related quality of life) OR abstract:(oral health related quality of life)) OR (title:(OHRQoL) OR abstract:(OHRQoL))))))	49
Biblioteca Virtual en Salud (BVS) -	(Calidad de vida relacionada con la salud bucal) AND (Hipomineralización Molar Incisivo), (Calidad de vida relacionada con la salud oral) AND (Hipomineralización Molar Incisivo), (Calidad de vida relacionada con la salud oral) AND (Hipomineralización Incisivo Molar) y (Calidad de vida relacionada con la salud bucal) AND (Hipomineralización Incisivo Molar)	12
Dentistry & Oral Sciences Source y	(oral health related quality of life) OR (oral health-related quality of life) OR (OHRQoL) OR (ohrqol) AND (Molar-Incisor Hypomineralization) AND (MIH).	12
Otras fuentes	Estudios extraídos de referencias estudios	6
Total		95

### Criterios de inclusión

Se incluyeron artículos originales publicados en revistas arbitradas por pares, provenientes de estudios primarios observacionales de cualquier diseño (cohortes, casos y controles, estudios antes-después (pre-post) con y sin grupo de control y estudios transversales) en población de 6 a 14 años con diagnóstico de HIM de acuerdo con los criterios del DDE *index* (FDI Working Group 1992) ^20^ o EAPD *index* (European Academy of Paediatric Dentistry 2003) ^21^. La CVRSB debió ser medida con escalas validadas como el *Child Perception Questionnaire* (CPQ), el *Parental-Caregiver Perception Questionnaire* (P-CPQ) o el *Child Oral Health Impact Profile* (COHIP). Asimismo, que fueran publicaciones en inglés o español.

### Criterios de exclusión

Publicaciones de revisiones sistemáticas, reportes de casos, cartas al editor, estudios de CVRSB en niños y adolescentes con otros DDE, publicaciones sin reporte de CVRSB. 

### Selección de los estudios

Tres autores (CAR, LLC, MJM), de manera independiente, examinaron los títulos y resúmenes del grupo de referencias obtenidas en la identificación de las bases de datos con la finalidad de excluir publicaciones duplicadas e inadecuadas para el fin de estudio. Después de la lectura del texto completo, discusión, retroalimentación y acuerdo, los artículos que no cumplían con los criterios de inclusión fueron eliminados. 

### Extracción de datos

Dos autores (CAR, LLC) leyeron el texto completo de los posibles artículos para su inclusión en la síntesis. En cada artículo elegible los autores extrajeron independientemente los datos de primer autor, año de publicación, país, diseño del estudio, tamaño de muestra, grupos de observación (número de casos, número de controles/grupo de comparación), edad de la población de estudio, niños (n y %), instrumento de medición de CVRSB, persona que responde el cuestionario de CVRSB, presencia y severidad de HIM (n y %), y descripción del hallazgo. 

### Evaluación de la calidad de los estudios

Se emplearon las herramientas de evaluación de calidad de estudios observacionales de cohortes y transversales “*Quality Assessment Tool for Observational Cohort and Cross-Sectional Studies*” y para estudios antes y después “*Quality Assessment Tool for Before-After (Pre-Post) Studies With No Control Group*” dependiendo del diseño del estudio (disponibles en: https://www.nhlbi.nih.gov/health-topics/study-quality-assessment-tools). 

## RESULTADOS

**Figura 1:**
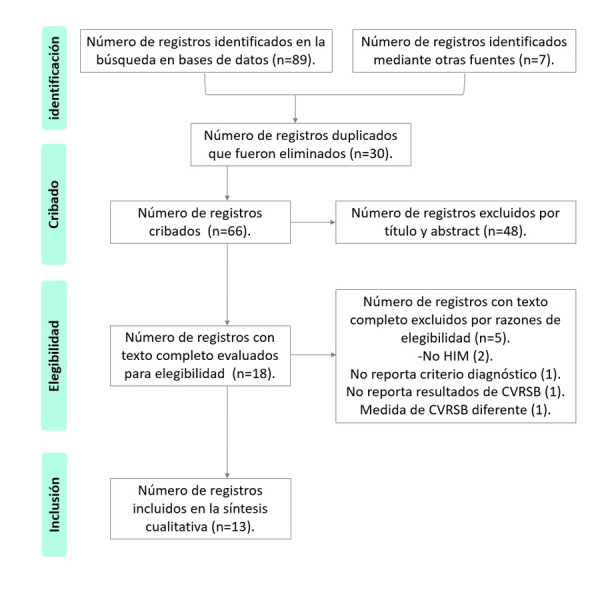
Diagrama de flujo del proceso de selección de los estudios

De la búsqueda en las bases electrónicas y las referencias de los estudios, se recuperó un total de 89 publicaciones y de las referencias de otros estudios se identificaron siete artículos. Posterior a la eliminación de referencias duplicadas y la evaluación manual del título y resumen se obtuvieron 18 artículos para lectura completa. Después de la lectura, evaluación de la calidad metodológica (Tabla 2) y discusión, cinco estudios se excluyeron debido a que evaluaron otro DDE como exposición, no reportaban el criterio diagnóstico de HIM o no informaron la evaluación de la CVRSB en los resultados, lo que derivó en un total de 13 considerados elegibles para la síntesis cualitativa (Figura 1).

**Tabla 2:** Evaluación de la calidad del estudio incluidos

Primer autor. Año de publicación	Diseño del estudio	Preguntas													
		1	2	3	4	5	6	7	8	9	10	11	12	13	14
Altner, 2022	Antes-después	S	S	N	NA	S	S	S	NA	S	S	N	NA		
Bekes, 2021	Antes-después	S	S	N	S	S	S	S	S	S	S	S	NA		
Dantas-Neta, 2016	Transversal	S	S	S	S	S	S	NA	S	S	NA	S	NA	NA	S
Dias, 2021	Transversal	S	S	S	S	S	S	NA	S	S	NA	S	NA	NA	S
Freitas Fernandes, 2021	Transversal	S	S	S	S	S	S	NA	S	S	NA	S	NA	NA	S
Gutiérrez, 2019	Transversal	S	S	S	S	S	S	NA	S	S	NA	S	NA	NA	S
Hasmun, 2020	Antes-después	S	S	S	S	S	S	NA	S	S	NA	N	NA		
Joshi, 2022	Transversal	S	S	S	S	S	S	NA	S	S	NA	S	NA	NA	N
Elhennawy, 2022	Transversal	S	S	S	S	S	S	NA	S	S	NA	S	NA	NA	N
Michaelis, 2021	Transversal	S	S	S	S	S	S	NA	S	S	NA	S	NA	NA	N
Portella, 2019	Transversal	S	S	S	S	S	S	NA	S	S	NA	S	NA	NA	S
Velandia, 2018	Transversal	S	S	N	S	S	S	NA	S	S	NA	S	NA	NA	N
Tugcu, 2022	Antes-después	S	S	N	S	N	S	S	NA	S	S	N	NA		
Vanhée, 2022	Transversal	S	S	S	S	N	S	NA	N	N	NA	S	NA	NA	N
Large, 2020	Transversal	S	S	N	S	N	S	NA	S	N	NA	S	NA	N	N

S: Sí cumple; N: No cumple; NA: No aplica.

Fuente: Elaboración propia. El enunciado de las preguntas está disponible en las herramientas en línea en: https://www.nhlbi.nih.gov/health-topics/study-quality-assessment-tools.

### Diseño y poblaciones de los estudios

La mayoría de los estudios se hicieron en Europa y los países donde más estudios se reportaron fueron Alemania y Brasil, con cinco y cuatro estudios respectivamente (Tabla 3).

**Tabla 3:** Datos extraídos de los estudios incluidos

Primer autor, año de publicación (país)	Diseño del estudio	N	CD	Gravedad de HIM n (%)			Grupos de estudio	Población de estudio		CVRSB	Descripción de los hallazgos	Puntajes totales y por dimensiones
				Leve	Moderada	Grave	n Grupo HIM (n Grupo comparación)	Edad	Niños n (%)	Instrumento de CVRSB				
Altner, 2022 (Alemania) (^24^)	Antes-después con grupo de comparación	210	EAPD	NR	NR	NR	105 (Grupo con caries 105)	7-11	107 (51)	CPQ 8-10	Los pacientes con HIM mostraron una puntuación CPQ total significativamente más alta en comparación con el grupo con caries. Las dimensiones de Limitación funcional y Bienestar emocional se evidenciaron significativamente más afectadas en el grupo con HIM.	Puntaje total Grupo HIM: 17,88 DE = 10,60; Grupo caries: 13,88 DE = 14,23. (p valor = 0,022) Dimensiones Grupo HIM: SO = 6,36* DE = 2,99; LF = 4,90 DE = 3,33; BE = 5,06* DE = 4,01; BS = 1,55 DE = 2,60. Grupo caries SO = 6,54 DE = 4,30; LF = 2,97 DE = 3,64; BE = 2,83 DE = 3,89; BS = 1,53 DE = 3,46
Bekes, 2021 (Austria/Alemania) (^21^)	Antes - después sin grupo de comparación	38	EAPD	NR	NR	NR	38	6-10	20 (52,6)	CPQ 8-11	La dimensión más afectada fue Síntomas orales, seguido de Limitación funcional y Bienestar emocional.	Puntaje total 14,7 DE = 5,9. Dimensiones SO = 7,5* DE = 3,0; LF = 4,5* DE = 1,6. BE = 1,8 DE = 1,9. BS = 0,9 DE = 1,9
Dantas-Neta, 2016 (Brasil) (^30^)	Transversal	594	EAPD	54 (49,5%)	30 (27,5%)	25 (23,0%)	109	11-14	No reporta	CPQ 11-14 SF / PCPQ-SF	Los niños con HIM grave tuvieron una tasa de impacto mayor en las dimensiones de síntomas orales y limitación funcional en comparación con los que no tenían HIM. Según la percepción de los padres, los niños con HIM grave tenían mayor puntaje que aquellos sin HIM en la dimensión de limitación funcional.	Puntaje total P CPQ: 7,26 DE = 6,84 Dimensiones SO = 1,86 DE = 2,00; LF = 2,67* DE = 2,76; BE = 2,75* DE = 3,67; BS = 7,26; 6,84 Puntaje total CPQ: 11,92 DE = 7,98 Dimensiones SO = 4,08* DE = 2,40; LF = 3,36* DE = 2,76; BE = 2,52; DE = 2,76; BS = 1,95; DE = 2,31
Dias, 2020 (Brasil) (^36^)	Transversal	253	EAPD	238 (94,1)	NR	15 (5,9)		6-12	125 (49,4)	CPQ 8-10 / CPQ 11-14 / PCPQ SF	Según la percepción de los padres la HIM severa se asoció con un mayor impacto en las dimensiones de Limitación funcional y Bienestar emocional. De acuerdo con la percepción de niños, la HIM severa no se asoció con mayores impactos en ninguna dimensión del CPQ ni en sus puntuaciones totales.	Puntaje total P CPQ 22,18 DE = 15,31. Dimensiones SO = 6,13 DE = 3,72; LF = 6,30* DE = 5,12; BE = 6,53* DE = 6,48; BS = 3,22 DE = 3,95 Puntaje total CPQ 8-10 15,11 DE = 10,93 Dimensiones SO = 5,52* DE = 3,19; LF = 3,31 DE = 3,25*; BE = 3,07 DE = 3,57; BS = 3,21 DE = 4,47 Puntaje total CPQ 11-14 22.95 DE = 14,45 Dimensiones SO = 6,78* DE = 3,58; LF = 7,06* DE = 5,31; BE = 4,24 DE = 4,23; BS = 4,87 DE = 5,36
Freitas Fernandes, 2021(Brasil) (^35^)	Transversal	463	EAPD	22 (44,0)	NR	28 (56,0)	50 (413)	11-14	170 (36,7)	CPQ 11-14 SF/ PCPQ-SF	Según la percepción de los niños la HIM no se asoció en la CVRSB. En contraste la HIM se asoció sólo con un mayor impacto en la dimensión de Bienestar Emocional según la percepción de los padres/cuidadores.	No reporta
Gutiérrez, 2019 (México) (^17^)	Transversal	411	EAPD	28 (6,8)	138 (33,6)	NR	166 (245)	8-10	217 (52,8)	CPQ 8-10	Los escolares con HIM experimentaron una mayor tasa de impacto negativo en comparación con los escolares sin HIM en el puntaje total (RR = 2,07 IC95% 2,00 - 2,14) y los cuatro dimensiones del CPQ 8-10. Asimismo, la severidad de la HIM tuvo un gradiente incremental donde a mayor severidad se evidenció mayor puntuación tanto en el puntaje total como en las cuatro dimensiones.	Puntuación total Grupo HIM: 58,2 DE = 30,3 Sin HIM Total = 27,6 DE = 28,0; SO = 6,0 DE = 5,9; LF = 4,8 DE = 6,2; BE = 5,5 DE = 6,4; BS = 8,8 DE = 13,0 Dimensiones SO = 11,1* DE = 61 LF = 11,9* DE = 7,2; BE = 11,1 DE = 6,9; BS = 24,0 DE = 14,4 Severidad leve Total = 52,6 DE = 31,5; Leve SO = 10,1* DE = 6,6; LF = 9,2 DE = 7,9; BE = 9,4 DE = 7,8; BS = 22,7* DE = 15,7 Severidad Moderada/Grave Total = 59,4 DE = 28,9; SO = 11,3* DE = 6,1; LF = 11,2 DE = 7,0; BE = 11,4 DE = 6,7; BS = 24,3* DE = 14,2
Hasmun, 2020 (Reino Unido) (^22^)	Antes - después sin grupo de comparación	103	NR	NR	NR	NR	108	7-16	35 (40,7)	COHIP-19	La puntuación total media de COHIP-SF19 aumentó significativamente después del tratamiento, lo que indica una mejora significativa CVRSB.	Puntaje total inicial 47,4 DE = 9,34. Puntaje total después del tratamiento 59,8 DE = 9,7
Joshi, 2022 (Alemania) (^27^)	Transversal	188	EAPD	índice (HIM/TNI)	índice (HIM/TNI)	índice (HIM/TNI)	94 (94)	8-10	107 (56,9)	CPQ 9-10	Las puntuaciones medias de impacto sobre la CVRSB totales y por dimensiones fueron significativamente más altas en los niños afectados por HIM comparados con las de niños sanos. Las puntuaciones del CPQ aumentaban de acuerdo a la severidad de la HIM.	Grupo con HIM Puntaje total Grupo con HIM: 13,88 DE = 8,91. Grupo sin HIM: 4,20 DE = 3,74 Dimensiones Grupo HIM SO = 6,88* DE = 3,76; LF = 2,35 DE = 2,36; BE = 2,91* DE = 3,23; BS = 1,72 DE = 2,62. Grupo sin HIM SO = 2,29 DE = 2,60; LF = 0,88 DE = 1,35; BE = 0,66 DE = 1,24; BS = 0,38 DE = 0,96
Elhennawy (Alemania) (^28^)	Transversal	317	EAPD	60	NR	157	217 (100)	7-14	212 (52)	COHIP-19	El grupo con HIM tuvo un puntaje significativamente menor que el grupo control lo que indica una peor CVRSB. El grupo con HIM severa tuvo significativamente menor puntaje en el total de CVRSB y en la dimensión socioemocional, escolar y de autoimagen.	Puntaje total Grupo con HIM: 60,7 DE = 10 Grupo sin HIM = 67,9 DE = 10,4 Dimensiones Grupo HIM Salud Oral = 12,8 DE = 3,4; Bienestar funcional = 13,6* DE = 2,7; Bienestar Socioemocional = 34,3* DE = 6,7. Grupo sin HIM Salud Oral = 16 DE = 3; Bienestar funcional = 14,9 DE = 2,1; Bienestar Socioemocional = 37 DE = 4,3
Michaelis 2021 (Alemania) (^29^)	Transversal	528	EAPD	88 (16,66)	88 (16,66)	88 (16,66)	264 (264)	7-10	266 (50,4)	CPQ 8-10	Los pacientes con HIM mostraron una puntuación media del CPQ significativamente mayor en comparación con el grupo de caries. La puntuación aumentó linealmente desde la categoría de baja severidad a alta severidad en ambos grupos, aunque la puntuación del grupo con HIM se mostró ligeramente mayor.	Puntuación total Grupo HIM: 10,7. Dimensiones SO = 3,8*; LF = 2,2; BE = 3,5*; BS = 0,9 Severidad Leve SO = 1,4*; LF = 0,1; BE = 2,9*; BS = 0,8; Total = 5,2 Moderada SO = 3,6* LF = 2,5 BE = 2,7* BS = 0,5; Total = 9,3 Grave SO = 6,4* LF = 4,9 BE = 5,0* BS = 1,3; Total = 17,7 Puntuación total Grupo caries: 8,1. Dimensiones SO = 4,8; LF = 1,3; BE = 1,5; BS = 0,5 Severidad Leve SO = 3,3; LF = 0,2; BE = 0,6; BS = 0,0; Total = 2,2 Moderada SO = 3,9; LF = 0,3; BE = 0,5; BS = 0,0; Total = 4,5 Severa SO = 5,9; LF = 2,4; BE = 2,3; BS = 0,7; Total = 11,9
Portella 2019 (Brasil) (^16^)	Transversal	728	EAPD	63 (8,7)	25 (3,4)	NR	88 (640)	8	372 (51,09)	CPQ 8-10	La HIM solo se asoció con mayor impacto en la dimensión de Síntomas Orales.	NR
Tugcu 2022 (Turquía) (^23^)	Antes - después sin grupo de comparación	55	EAPD	NR	NR	NR	55	11-14	16 (40)	CPQ 11-14	Se evidenció una mejora significativa en la CVRSB después del tratamiento. La dimensión más afectada antes del tratamiento fue Bienestar emocional, seguido de síntomas orales. Después de la terapia todos los puntajes de las dimensiones disminuyeron significativamente.	Puntaje total 33,27 DE 16,46. Dimensiones SO = 9,20* DE 4,24; LF = 7.20 DE 5,03; BE = 11,87* DE 8,73; BS = 5,0 DE 6,0
Velandia 2018 (Colombia) (^26^)	Transversal	88	EAPD	20 (45,4)	24 (54,6)	NR	44 (44)	7-10	41 (46,5)	CPQ 8-10	Todas las puntuaciones del CPQ 8-10 total y por dimensiones fueron significativamente mayores en el grupo con HIM.	Puntaje total Grupo HIM = 12,5 RIQ = 17 Grupo sin HIM = 4,0 RIQ = 3,5 Dimensiones Grupo HIM: SO = 5,0* RIQ = 3,0; LF = 2,0 RIQ = 4,5; BE = 4,0* RIQ = 6,5; BS = 2,0 RIQ = 5,5. Grupo sin HIM: SO = 1,0 RIQ = 1,0; LF = 0,0 RIQ = 1,0; BE = 0,0 RIQ = 1,01; BS = 0,0 RIQ = 2,0

N: Tamaño de muestra. CD: Criterios diagnóstico. NR: No reportado. HIM: Hipomineralización incisivo molar. EAPD: European Academy of Paediatric Dentistry. TNI: *Treatment need index*. CVRSB: Calidad de Vida Relacionada con la Salud Bucal. CPQ: *Child Perception Questionnaire*. P-CPQ: *Parental-Caregiver Perception Questionnaire*. COHIP: *Child Oral Health Impact Profile*. SF: Short form. SO: Síntomas orales. LF: Limitación funcional. BE: Bienestar emocional. BS: Bienestar social. DE: Desviación estándar. RIQ: Rango intercuartil. *: Puntuación mayor entre las dimensiones.

La mayoría de los estudios se ejecutaron con diseño transversal. Tres fueron estudios antes-después (pre-post) sin grupo de comparación ^21^^-^^23^ y un estudio antes-después incluyó un grupo de comparación integrado por niños con caries ^24^. Por otro lado, ocho estudios transversales incluyeron grupos de comparación, siendo estos seis reportes en niños sin HIM ^17^^,^^25^^-^^28^ y dos estudios en niños con caries ^24^^,^^29^.

En relación con el criterio diagnóstico, el más frecuente fue el EAPD, que se usó en 12 estudios (Tabla 3). Solo un estudio no informó aspectos del índice diagnóstico, dado que reportó que los participantes fueron remitidos de un centro de atención con diagnóstico previo ^22^.

Las edades de los participantes fueron variables y estuvieron comprendidas en un rango de 6 a 16 años. La mayor proporción de estudios se hicieron en niños entre los 7 y 16 años (Tabla 3). 

De 13 estudios, nueve informaron la severidad de la HIM, de los cuales solo dos informaron la gravedad en términos de “leve, moderada y severa” ^16^^,^^31^.

En ocho de los 13 estudios se usó el CPQ en alguna de sus versiones para medir la CVRSB en los niños, siendo las versiones CPQ 8-10 ^31^ y CPQ 11-14 ^32^ las más utilizadas. Por otra parte, tres estudios midieron la CVRSB en niños y padres o cuidadores conjuntamente usando la escala P-CPQ ^33^ en su forma abreviada de 14 y 16 preguntas, mientras que el COHIP-19 ^34^ se utilizó en dos estudios (Tabla 3). En la mayoría de los estudios, los respondedores fueron los niños. Solo en tres estudios se evaluó la percepción de los padres (Tabla 1) y en ningún informe se consideró solo la percepción de los padres como indicador del impacto de la HIM sobre la CVRSB.

### Calidad de los estudios

Dentro de las fortalezas metodológicas de los estudios se destaca que sólo cinco estudios de 13 reportaron ajuste de los factores de confusión mediante análisis multivariado ^16^^,^^17^^,^^30^^,^^35^^,^^36^. Conviene subrayar que todos usaron como técnica del análisis multivariado la regresión de Poisson con varianza robusta. 

Por otra parte, únicamente cuatro estudios informaron el proceso de calibración (estandarización) de los evaluadores que realizaron el examen clínico para el diagnóstico de HIM ^16^^,^^17^^,^^30^^,^^35^^,^^36^.

### Asociación entre la CVRSB y la HIM

Las dimensiones en las que se reportaron los mayores puntajes promedios fueron “Síntomas orales” y “Bienestar emocional”, independientemente de la medida empleada.

Diez de los 13 reportaron asociación de la HIM con un mayor impacto en al menos una de las dimensiones de CVRSB. Por otra parte, tres estudios encontraron mayor impacto en todas las dimensiones ^17^^,^^37^^,^^38^.

Tres de los 13 estudios informaron un impacto significativamente mayor en las dimensiones “Limitación funcional” ^24^^,^^30^^,^^36^, “Síntomas orales” ^16^^,^^30^^,^^36^ y “Bienestar emocional” ^24^^,^^35^^,^^36^.

Cuando la CVRSB fue evaluada bajo la percepción de los niños las dimensiones más afectadas fueron “Síntomas orales” y “Bienestar emocional”. En cambio, según los padres o cuidadores, las dimensiones más afectadas fueron “Limitación funcional” y “Bienestar emocional”. En los estudios en los que la CVRSB se evaluó conjuntamente en niños y padres, según los niños, las dimensiones más afectadas fueron “Síntomas orales” y “Limitación funcional”.

Aunque el objetivo de esta revisión no fue determinar si las intervenciones clínicas mejoran la CVRSB en niños, se encontró que en los cuatro estudios antes-después los pacientes mejoraron después del tratamiento (Tabla 3).

Solo cuatro estudios evaluaron la CVRSB de niños con HIM y su relación con la edad ^22^^,^^24^^,^^28^^,^^29^; no obstante, no se encontraron asociaciones significativas entre estas variables.

### Impacto de acuerdo con el sexo

De cinco estudios que reportaron análisis multivariado, cuatro reportaron una peor CVRSB en las niñas con HIM después del ajuste por los posibles factores de confusión ^16^^,^^17^^,^^30^^,^^35^. Señalando que el impacto general de la HIM sobre la CVRSB en las niñas fue entre un 14% y un 16% mayor en comparación con los niños. En el análisis por dimensiones, se identificó que en “Síntomas orales” el impacto fue entre un 4% y un 12% mayor en las niñas, y que la dimensión “Limitación funcional” se vio más afectada entre un 18% y un 58% en las niñas. Finalmente, el impacto en la dimensión “Bienestar emocional” en las niñas fue un 58% mayor.

## DISCUSIÓN

### Calidad de vida relacionada con la salud bucal

El estudio de la CVRSB y la HIM representa un interés por fenómenos relativamente nuevos y que se han incrementado en las últimas décadas, he ahí una posible explicación en que se hayan recuperado pocos estudios de la búsqueda bibliográfica. No obstante, este particular interés queda evidenciado en que la mayoría de los estudios fueron realizados recientemente. Indiscutiblemente, este factor se relaciona con que sean pocos los estudios de revisión sobre el impacto de los DDE y aún menos de la HIM sobre la CVRSB, ya que, a nuestro entender, esta es la segunda revisión que aborda el tema. 

En relación, con otras revisiones sistemáticas, estos hallazgos difieren de lo reportado por Jälevik *et al*. ^18^ en cuanto a la región de ejecución de los estudios, ya que, en esa revisión se encontró que en los países de América Latina fue donde más se habían realizado investigaciones. Esto obedece a que se ha publicado nueva evidencia realizada en países europeos después de la fecha en la que se realizó la búsqueda bibliográfica del estudio de Jälevik. 

El rango de edad de los participantes también difirió con la investigación de la revisión anterior, mientras que en sus resultados el rango de 8-10 años fue la edad más común. Por otro lado, la proporción de estudios que informaron la severidad de la HIM fue similar entre ambos estudios ^19^.

Las dimensiones de “Síntomas orales” y “Limitación funcional” han sido reportadas por Jälevik B ^19^ como las más afectadas. Esto difiere parcialmente con nuestros hallazgos, puesto que se encontró mayor impacto en “Bienestar emocional” y “Síntomas orales”. No es sorprendente que esta última dimensión sea una de las más afectadas, puesto que se ha indicado desde la base fisiopatológica de la HIM, se genera una respuesta inflamatoria del órgano pulpar, derivando en dolor e hipersensibilidad, producto de la sostenida infiltración bacteriana en la estructura hipomineralizada del esmalte dental ^39^^-^^42^.

En relación con el mayor impacto de la HIM sobre la dimensión de “Bienestar emocional”, se puede explicar en que la forma más común de HIM es la que afecta molares e incisivos, sobre todo en la superficie vestibular, como pasa en otros DDE. Por consiguiente, es posible que, ante el deterioro en la estética dental, los niños se sientan renuentes a sonreír o hablar, lo que acentúa su inseguridad a esta edad y compromete su bienestar emocional ^43^. Asimismo, estudios previos como el de Coffield *et al*. ^44^, que han evaluado otros DDE como la amelogénesis imperfecta (AI) y su relación con aspectos psicosociales, señalan que los sujetos afectados tienen baja autoestima y mayor miedo a la evaluación negativa por parte de otras personas. 

El hecho de que las niñas percibieran mayor afectación podría explicarse por una mayor preocupación de estas en torno a problemas de salud relacionados con la función y la estética ^45^^,^^46^.

### Aspectos metodológicos de los estudios

Respecto al análisis multivariado, se destaca que todos los estudios que ajustaron factores de confusión usaron una técnica de regresión de Poisson con varianza robusta. Esta es una alternativa adecuada cuando la variable dependiente a evaluar es un conteo ^47^, como es el caso de las puntuaciones de las escalas de CVRSB. 

Es una práctica común que los estudios de CVRSB informen resultados binarios (Mejor CVRSB/Peor CVRSB). Sin embargo, se sugiere que este método presenta desventajas operativas como el establecimiento de un punto de corte arbitrario que puede resultar en pérdida de información y poder estadístico reducido para evaluar las covariables ^48^^,^^49^. No obstante, su amplia utilización puede deberse a que permite mayor facilidad para los clínicos y personas interesadas entender en qué proporción algún factor modifica la probabilidad de afectar la CVRSB. 

### Implicaciones para la investigación

A pesar de que casi todos los estudios tenían una metodología bien definida y los resultados fueron reportados en medidas de resumen similares, lo que hizo posible la comparación de los impactos en la CVRSB y sus dimensiones, la mayor proporción fueron de diseño transversal, lo cual es una limitación para indicar una relación causal confiable entre la CVRSB deteriorada por la HIM, así como la falta de seguimiento a los pacientes que permitan aproximaciones longitudinales. Por ello, se sugieren diseños observacionales más robustos, como los estudios de cohortes y, para los casos de intervenciones terapéuticas, diseños de ensayos clínicos controlados para generar resultados más confiables y comparables. 

También es recomendable considerar ajustar factores de confusión como la caries y su severidad, la gravedad de la MIH, el número y tipo de dientes afectados, la presencia de maloclusiones y el uso de aparatología ortopédica maxilar y ortodóntica en los afectados con HIM, ya que se han reportado como factores asociados a peor CVRSB en niños ^50^^,^^51^.

El presente trabajo proporciona una base de evidencia con estudios seleccionados de calidad metodológica considerable, que puede ayudar a los odontólogos en la incorporación de la CVRSB en su práctica clínica. Esto reforzará el abordaje integral de los pacientes infantiles y permitirá sensibilizar sobre aspectos adicionales a los signos y síntomas de la HIM, como los emocionales y sociales, que no son comúnmente abordados en la anamnesis convencional.

También puede ayudar a orientar el tratamiento interdisciplinar en aras de facilitar la comprensión de las necesidades estéticas, funcionales, de autoestima y socialización de los niños y adolescentes afectados con HIM.

## CONCLUSIONES

Con base en los artículos seleccionados, se concluye que el efecto negativo de la HIM sobre la CVRSB en niños y adolescentes de 6-17 años fue significativo en las dimensiones “Síntomas orales” y “Bienestar emocional” cuando se empleó el CPQ como medida. Mientras que, al utilizar el instrumento el P-CPQ, el impacto de la HIM se reflejó mayormente en las dimensiones “Limitación funcional” y “Bienestar emocional”. 

Las niñas con HIM parecen tener una peor CVRSB en comparación con niños afectados. A pesar de que el auge de los estudios de CVRSB va en ascenso, existe escasez de reportes con buena calidad metodológica que estudien el impacto de la HIM en la CVRSB de los infantes.

## Financiación:

Esta investigación fue autofinanciada

## References

[1] Subramaniam P, Gupta T, Sharma A (2016). Prevalence of molar incisor hypomineralization in 7-9-year-old children of Bengaluru City, India. Contemp Clin Dent.

[2] Gómez Clavel JF, Amato Martínez D, Trejo Iriarte CG, García Muñoz A (2018). Análisis de la relación entre la hipomineralización incisivo molar y los factores asociados a su etiología. Rev. Odont. Mex.

[3] Félix FM (2020). Defining the prevalence of molar incisor hypomineralization in Brazil. Pesquisa Brasileira em Odontopediatria e Clínica Integrada.

[4] Reyes MR (2019). Demarcated opacity in primary teeth increases the prevalence of molar incisor hypomineralization. Brazilian Oral Research.

[5] Buchgraber B, Kqiku L, Ebeleseder KA (2018). Molar incisor hypomineralization proportion and severity in primary public school children in Graz, Austria. Clin Oral Investig.

[6] Rai A, Singh A, Menon I, Singh J, Rai V, Aswal GS (2018). Molar incisor hypomineralization prevalence and risk factors among 7-9 years old school children in Muradnagar, Ghaziabad. Open Dent J.

[7] Villanueva-Gutiérrez T, Irigoyen-Camacho ME, Castaño-Seiquier A, Zepeda-Zepeda MA, Sanchez-Pérez L, Frechero NM (2019). Prevalence and severity of molar-incisor hypomineralization, maternal education, and dental caries a cross-sectional study of mexican schoolchildren with low socioeconomic status. J Int Soc Prev Community Dent.

[8] Zhao D, Dong B, Yu D, Ren Q, Sun Y (2018). The prevalence of molar incisor hypomineralization evidence from 70 studies. Int J Paediatr Dent.

[9] Parekh S, Almehateb M, Cunningham SJ (2014). How do children with amelogenesis imperfecta feel about their teeth. Int J Paediatr Dent.

[10] Craig SA, Baker SR, Rodd HD (2015). How do children view other children who have visible enamel defects. Int J Paediatr Dent.

[11] SALGADO AO, PERALVO V, TORRES A, MATEOS MV, RIBAS D, CASTANO A (2016). Prevalencia del síndrome de hipomineralización incisivo-molar revisión de la literatura. Odontol Pediátr.

[12] da Costa-Silva CM, Jeremias F, de Souza JF, Cordeiro RC, Santos-Pinto L, Zuanon AC (2010). Molar incisor hypomineralization prevalence, severity and clinical consequences in Brazilian children. Int J Paediatr Dent.

[13] Resende P, Oliveira Favretto C (2019). Desafios clínicos no tratamento de hipomineralização molar incisivo. Journal of Oral Investigations.

[14] Elhennawy K, Schwendicke F (2016). Managing molar-incisor hypomineralization A systematic review. J Dent.

[15] Raposo F, de Carvalho Rodrigues AC, Lia ÉN, Leal SC (2019). Prevalence of hypersensitivity in teeth affected by molar-incisor hypomineralization (MIH). Caries Res.

[16] Portella PD, Menoncin BLV, de Souza JF, de Menezes JVNB, Fraiz FC, Assuncao LRDS (2019). Impact of molar incisor hypomineralization on quality of life in children with early mixed dentition A hierarchical approach. Int J Paediatr Dent.

[17] Gutiérrez TV, Ortega CCB, Pérez NP, Pérez AG (2019). Impact of molar incisor hypomineralization on oral health-related quality of life in mexican schoolchildren. J Clin Pediatr Dent.

[18] Jalevik B, Sabel N, Robertson A (2022). Can molar incisor hypomineralization cause dental fear and anxiety or influence the oral health-related quality of life in children and adolescents -a systematic review. Eur Arch Paediatr Dent.

[19] Liberati A, Altman DG, Tetzlaff J, Mulrow C, Gøtzsche PC, Ioannidis JP, Clarke M, Devereaux PJ, Kleijnen J, Moher D (2009). The PRISMA statement for reporting systematic reviews and meta-analyses of studies that evaluate health care interventions explanation and elaboration. PLoS Med.

[20] Weerheijm KL, Duggal M, Mejáre I, Papagiannoulis L, Koch G, Martens LC, Hallonsten AL (2003). Judgement criteria for molar incisor hypomineralisation (MIH) in epidemiologic studies: a summary of the European meeting on MIH held in Athens, 2003. Eur J Paediatr Dent.

[21] Bekes K, Amend S, Priller J, Zamek C, Stamm T, Kramer N (2021). Changes in oral health-related quality of life after treatment of hypersensitive molar incisor hypomineralization-affected molars with a sealing. Clin Oral Investig.

[22] Hasmun N, Vettore MV, Lawson JA, Elcock C, Zaitoun H, Rodd HD (2020). Determinants of children&apos;s oral health-related quality of life following aesthetic treatment of enamel opacities. J Dent.

[23] Tugcu N, Sezer B, Caliskan C, Durmus B, Kargul B (2022). Changes in oral health-related quality of life after treatment of molar incisor hypomineralization using Glass Hybrid Restorations. Journal of the Pakistan Medical Association.

[24] Altner S, Ebel M, Ritschl V, Stamm T, Hirsch C, Bekes K (2022). Treatment of severe caries and molar incisor hypomineralization and its influence on oral health-related quality of life in children a comparative study. Int J Environ Res Public Health.

[25] Freitas Fernandes LH, Laureano ICC, Farias L, Andrade NM, Soares Forte FD, Barros Alencar CR, Cavalcanti AL (2021). Incisor Molar Hypomineralization and Quality of Life A population-based study with brazilian schoolchildren. Int J Dent.

[26] Velandia LM, Álvarez LV, Mejía LP, Rodríguez MJ (2018). Oral health-related quality of life in Colombian children with Molar-Incisor Hypomineralization. Acta Odontol Latinoam.

[27] Joshi T, Rahman A, Rienhoff S, Rienhoff J, Stamm T, Bekes K (2022). Impact of molar incisor hypomineralization on oral health-related quality of life in 8-10-year-old children. Clin Oral Investig.

[28] Elhennawy K, Rajjoub O, Reissmann DR, Doueiri MS, Hamad R, Sierwald I, Wiedemann V, Bekes K, Jost-Brinkmann PG (2022). The association between molar incisor hypomineralization and oral health-related quality of life a cross-sectional study. Clin Oral Investig.

[29] Michaelis L, Ebel M, Bekes K, Klode C, Hirsch C (2021). Influence of caries and molar incisor hypomineralization on oral health-related quality of life in children. Clin Oral Investig.

[30] Dantas-Neta NB, Moura LF, Cruz PF, Moura MS, Paiva SM, Martins CC, Lima MD (2016). Impact of molar-incisor hypomineralization on oral health-related quality of life in schoolchildren. Braz Oral Res.

[31] Jokovic A, Locker D, Stephens M, Kenny D, Tompson B, Guyatt G (2002). Validity and reliability of a questionnaire for measuring child oral-health-related quality of life. J Dent Res.

[32] Jokovic A, Locker D, Tompson B, Guyatt G (2004). Questionnaire for measuring oral health-related quality of life in eight- to ten-year-old children. Pediatr Dent.

[33] Jokovic A, Locker D, Stephens M, Kenny D, Tompson B, Guyatt G (2003). Measuring parental perceptions of child oral health-related quality of life. J Public Health Dent.

[34] Broder HL, Wilson-Genderson M, Sischo L (2012). Reliability and validity testing for the Child Oral Health Impact Profile-Reduced (COHIP-SF 19). J Public Health Dent.

[35] Freitas Fernandes LH, Laureano ICC, Farias L, Andrade NM, Soares Forte FD, Barros Alencar CR, Cavalcanti AL (2021). Incisor Molar Hypomineralization and Quality of Life A Population-Based Study with Brazilian Schoolchildren. Int J Dent.

[36] Dias FMCS, Gradella CMF, Ferreira MC, Oliveira LB (2021). Molar-incisor hypomineralization parent&apos;s and children&apos;s impact perceptions on the oral health-related quality of life. Eur Arch Paediatr Dent.

[37] Joshi T, Rahman A, Rienhoff S, Rienhoff J, Stamm T, Bekes K (2022). Impact of molar incisor hypomineralization on oral health-related quality of life in 8-10-year-old children. Clin Oral Investig.

[38] Velandia LM, Álvarez LV, Mejía LP, Rodríguez MJ (2018). Oral health-related quality of life in Colombian children with Molar-Incisor Hypomineralization. Acta Odontol Latinoam.

[39] Americano GC, Jacobsen PE, Soviero VM, Haubek D (2017). A systematic review on the association between molar incisor hypomineralization and dental caries. Int J Paediatr Dent.

[40] da Cunha Coelho ASE, Mata PCM, Lino CA, Macho VMP, Areias CMFGP, Norton APMAP, Augusto APCM (2019). Dental hypomineralization treatment A systematic review. J Esthet Restor Dent.

[41] Elhennawy K, Manton DJ, Crombie F, Zaslansky P, Radlanski RJ, Jost-Brinkmann PG, Schwendicke F (2017). Structural, mechanical and chemical evaluation of molar-incisor hypomineralization-affected enamel A systematic review. Arch Oral Biol.

[42] Schwendicke F, Elhennawy K, Reda S, Bekes K, Manton DJ, Krois J (2019). Corrigendum to &raquo;,» &reg;,® &sect;,§ &shy;,­ &sup1;,¹ &sup2;,² &sup3;,³ &szlig;,ß &THORN;,Þ &thorn;,þ &times;,× &Uacute;,Ú &uacute;,ú &Ucirc;,Û &ucirc;,û &Ugrave;,Ù &ugrave;,ù &uml;,¨ &Uuml;,Ü &uuml;,ü &Yacute;,Ý &yacute;,ý &yen;,¥ &yuml;,ÿ &para;,¶ Global burden of molar incisor hypomineralization &raquo;,» &reg;,® &sect;,§ &shy;,­ &sup1;,¹ &sup2;,² &sup3;,³ &szlig;,ß &THORN;,Þ &thorn;,þ &times;,× &Uacute;,Ú &uacute;,ú &Ucirc;,Û &ucirc;,û &Ugrave;,Ù &ugrave;,ù &uml;,¨ &Uuml;,Ü &uuml;,ü &Yacute;,Ý &yacute;,ý &yen;,¥ &yuml;,ÿ &para;,¶ (J Dent. 68C (2018) 10-18).. J Dent.

[43] Chankanka O, Levy SM, Warren JJ, Chalmers JM (2010). A literature review of aesthetic perceptions of dental fluorosis and relationships with psychosocial aspects/oral health-related quality of life. Community Dent Oral Epidemiol.

[44] Coffield KD, Phillips C, Brady M, Roberts MW, Strauss RP, Wright JT (2005). The psychosocial impact of developmental dental defects in people with hereditary amelogenesis imperfecta. J Am Dent Assoc.

[45] Piovesan C, Antunes JL, Guedes RS, Ardenghi TM (2010). Impact of socioeconomic and clinical factors on child oral health-related quality of life (COHRQoL). Qual Life Res.

[46] Michel G, Bisegger C, Fuhr DC, Abel T, KIDSCREEN Group (2009). Age and gender differences in health-related quality of life of children and adolescents in Europe a multilevel analysis. Qual Life Res.

[47] Barros AJ, Hirakata VN (2003). Alternatives for logistic regression in cross-sectional studies an empirical comparison of models that directly estimate the prevalence ratio. BMC Med Res Methodol.

[48] Higgins JPT, Green S (2022). Cochrane Handbook for Systematic Reviews of Interventions Version Versión 6.3, 2022.

[49] Borges TS, Vargas-Ferreira F, Kramer PF, Feldens CA (2017). Impact of traumatic dental injuries on oral health-related quality of life of preschool children A systematic review and meta-analysis. PLoS One.

[50] Wall A (2020). Do malocclusions affect oral health related quality of life. Evid Based Dent.

[51] Alrashed M, Alqerban A (2021). The relationship between malocclusion and oral health-related quality of life among adolescents a systematic literature review and meta-analysis. Eur J Orthod.

